# Eugenol prevents fMLF-induced superoxide anion production in human neutrophils by inhibiting ERK1/2 signaling pathway and p47phox phosphorylation

**DOI:** 10.1038/s41598-019-55043-8

**Published:** 2019-12-06

**Authors:** Amina Chniguir, Coralie Pintard, Dan Liu, Pham My-Chan Dang, Jamel El-Benna, Rafik Bachoual

**Affiliations:** 1 Faculty of Sciences of Gabes, University of Gabes, Gabes, Tunisia; 2 Laboratory of Plant Improvement and Valorization of Agroresources, National School of Engineering of Sfax, Sfax, Tunisia; 3 INSERM U1149, CNRS ERL8252 Inflammation Research Center, Paris, France; 4 University of Paris Diderot, Sorbonne Paris City, Inflamex Laboratories, Faculty of Medicine, Xavier Bichat, Paris, France

**Keywords:** Chronic obstructive pulmonary disease, Chronic inflammation

## Abstract

Eugenol is a polyphenol extracted from *Syzygium aromaticum* essential oil. It is known to have anti-inflammatory and chemoprotective properties as well as a potent anti-oxidant activity due the presence of its phenolic group. In this study, we examined the effects of eugenol on neutrophil superoxide production, a key process involved in innate immunity and inflammation. Superoxide anion generationin human neutrophils was measured by cytochrome c reduction assay. Western blotting was used to analyze the phosphorylation of, p47phox, MAPKinases (p38 and ERK1/2), MEK1/2 and Raf, key proteins involved in the activation of NADPH oxidase. Pretreatment of neutrophils by increasing concentrations (2.5 µg/mL–20 µg/mL) of eugenol for 30 min, inhibited significantly (p < 0.001) superoxide anion generation induced by the chemotactic peptide formyl-Met-Leu-Phe (fMLF) with an IC50 of 5 µg/mL. Phorbolmyristate acetate (PMA)-stimulated O2^−^ production was affected only at the highest eugenol concentration (20 µg/mL). Results showed that eugenol decreased the phosphorylation of p47phox onSer-345 and Ser-328, the translocation of p47phox to the membranesand the phosphorylation of Raf, MEK1/2 and ERK1/2 proteins. Taken together, our results suggest that eugenol inhibits the generation of superoxide anion by neutrophils via the inhibition of Raf/MEK/ERK1/2/p47phox-phosphorylation pathway.

## Introduction

Natural exogenous molecules have been used as alternative therapeutic approaches for treating oxidative stress-related health problems, such as atherosclerosis, diabetes, cancer and inflammatory diseases^1^. These molecules are mainly derived from food and medicinal plants. A wide range of non-enzymatic antioxidants can be produced by plants and they have the ability to attenuate ROS-induced oxidative damage^2^. These natural molecules are especially polyphenols and carotenoids, exhibit various biological activities^3^. Eugenol (C_10_H_12_O_2_), a phenolic compound, is the major constituent of *Sysygium aromaticum* (L.) Merr. & L.M. Perry, Myrtaceae), it represents between 45 and 90% of the essential oil^4^. Eugenol is a colorless or a light yellowish fluid, slightly soluble in water and soluble in organic solvents^5^. As a natural product, eugenol has gained a great deal of attention in topical applications. Many reports indicate that it is endowed with many pharmacological activities including anti-bacterial^6^, anti-fungal^7^, local-anesthesic^8^, anti-tumoral^9^ and anti-inflammatory effect^10^. However, the effect of eugenol on inflammatory immune cells is less documented.

Polymorphonuclear neutrophils (PMN) are the first cells recruited to sites of infection or inflammation attracted by chemoattractants such as the complement fraction C5a, Interleukin-8, platelet activating factor and the bacterial peptide N-formyl-methionyl-leucylphenyl-alanine (fMLF)^11^. Their essential activity is the phagocytosis and subsequent killing of microorganisms^12^. Stimulated neutrophils producehigh amounts of reactive oxygen species (ROS) through the activation of NADPH oxidase complex^13^. This activation occurs through a complex series of protein interactionsleading to the assembly of membrane proteins (gp91phox, p22phox) and the cytosolic components (p40phox, p47phox, p67phox). Thus, catalyzing a rapid electron transfer from the NADPH via the complex to oxygen generating superoxide anion (O2^−^)^14^. Superoxide acts as the precursor of other reactive oxygen species (ROS) such as hydrogen peroxide (H_2_O_2_) and hypochlorous acid (HOCl) generated by heme enzyme myeloperoxidase^15^. At physiological concentrations, ROS are involved in the host defense response and acts as signaling molecules that regulate cell growth, adhesion toward other cells, differentiation, senescence, and apoptosis^16^. Further, enhanced ROS generation is considered central to the progression of inflammatory diseases^17^. The mechanisms implicated in the activation of NADPH oxidase are complex and diverse. However, two major steps are well established to be required for NADPH oxidase activation, the phosphorylation of p47phox on several serines and the translocation of the cytosolic subunits to the membrane^18^. In this context, our work aimed to study the effect of eugenol on neutrophil superoxide production and the signaling pathways implicated in this process.

## Results

### Eugenol strongly inhibits fMLF-induced superoxide anion production by neutrophils

**Figure 1:**
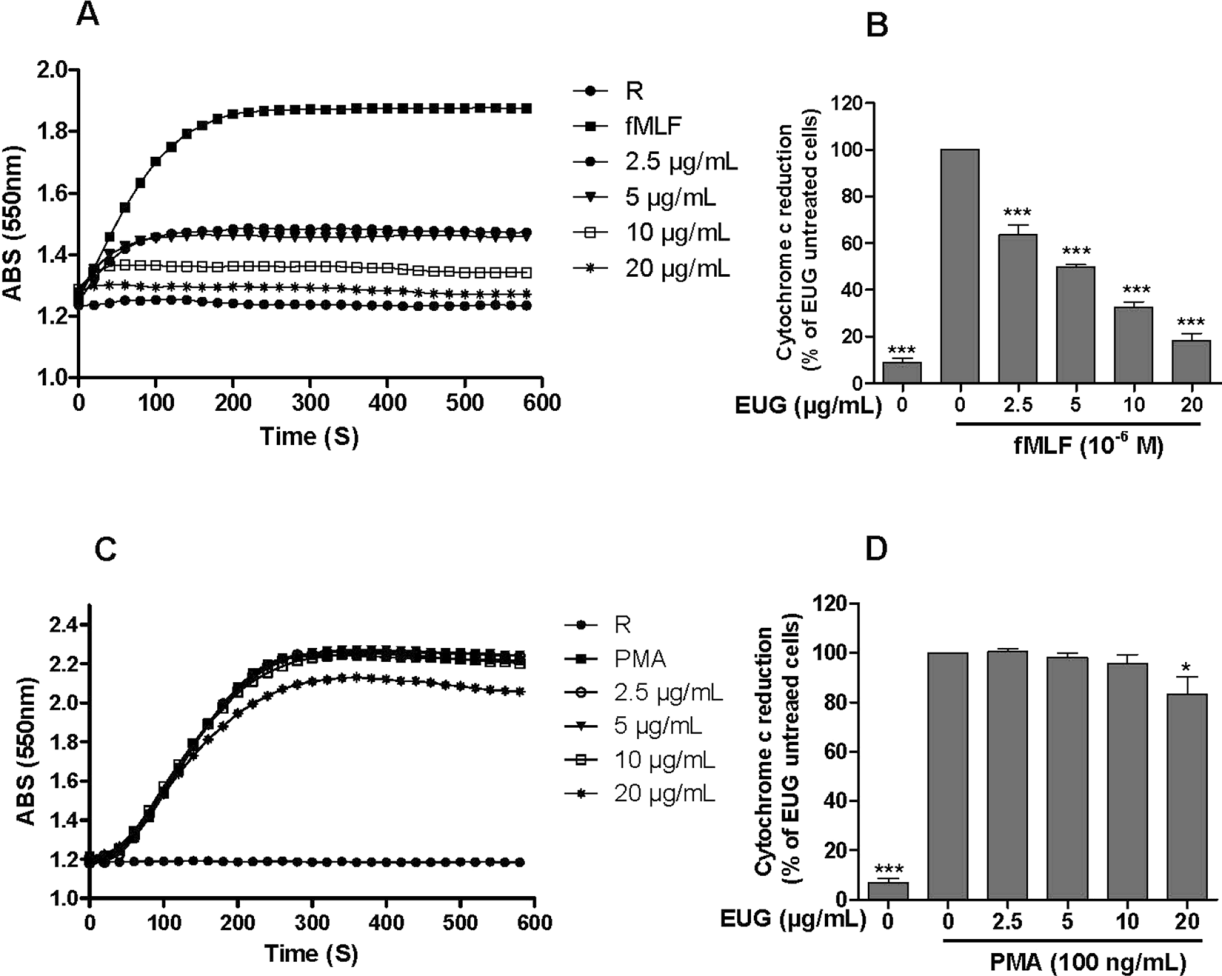
Eugenol strongly inhibits superoxide production by fMLF-stimulated human PMN. Human PMN (10^6^/ml) were incubated in the presence or absence of increasing concentrations of EUG for 30 min at 37 °C, then stimulated with fMLF (10^−6^ M) or with PMA (100 ng/mL). Superoxide anion production was measured by cytochrome c reduction assay at 550 nm. (**A**,**C**) Kinetics of cytochrome c reduction in the presence of increasing concentrations of EUG by fMLF- and PMA-stimulated PMN, respectively. (**B**,**D**) The initial velocity of the reaction from several experiments was expressed as percentage of control (PMN stimulated by fMLF or PMA). Data are expressed as mean ± SEM of 3 experiments: p < 0.005 as compared to control (EUG untreated cells; 100%). R: Resting cells.

Cyochrome c reduction assay is a specific technique for measuring the amounts of superoxide anion, first product of the NADPH oxidase. To investigate the effect of eugenol on superoxide anion production by human neutrophils, cells were incubated with increasing concentrations of eugenol for 30 min at 37 °C, then stimulated with fMLF or PMA and superoxide anion production was detected in the presence of cytochrome c by a spectrophotometer at 550 nm. Results showed that eugenol dose dependently (2.5 µg/mL–20 µg/mL) inhibited superoxide anion production by neutrophils stimulated by fMLF (Fig. 1A,B and supplementary file). This inhibition was statistically significant at low concentration of 2.5 µg/mL (p < 0.001) with IC_50_ value of 5 µg/mL. However, the inhibition was less important in neutrophils stimulated with Phorbolmyristate acetate (PMA), an activator of neutrophils functions via direct action on PKC (Fig. 1C,D). Results showed that superoxide anion production, by PMA-stimulated neutrophils, was reduced significantly (17%) with the highest concentration of eugenol (20 µg/mL).

### Eugenol is not toxic for human neutrophils

**Figure 2:**
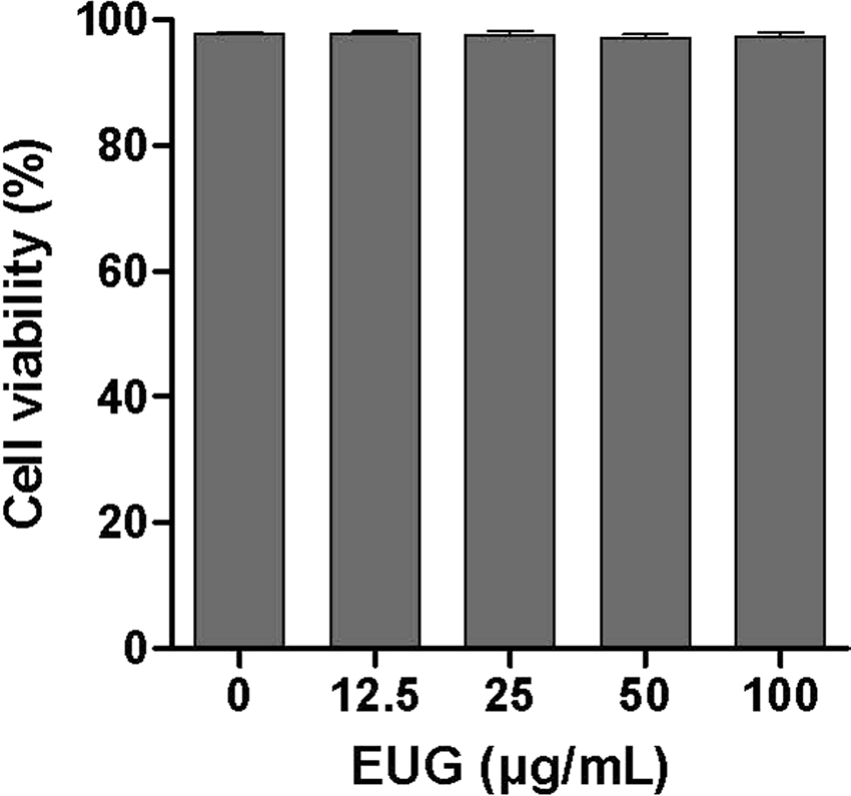
Cytotoxicity of eugenol. Human PMN were incubated in the presence or absence of increasing concentrations of eugenol for 30 min at 37 °C. Cell viability was assessed by trypan blue exclusion and results expressed as percentage of control untreated cells. Data are expressed as mean ± SEM of three or more separate experiments. p < 0.005 as compared to control (EUG untreated cells; 100%).

Cytotoxic effect of eugenol was determined by the decrease of viability of neutrophils using trypan blue dye test. As shown in Fig. 2, cell viability was not affected in different tested concentrations even at the highest dose of 100 µg/mL.

### Eugenol reduces p47phox-phosphorylation on Ser-328 and Ser-345

**Figure 3:**
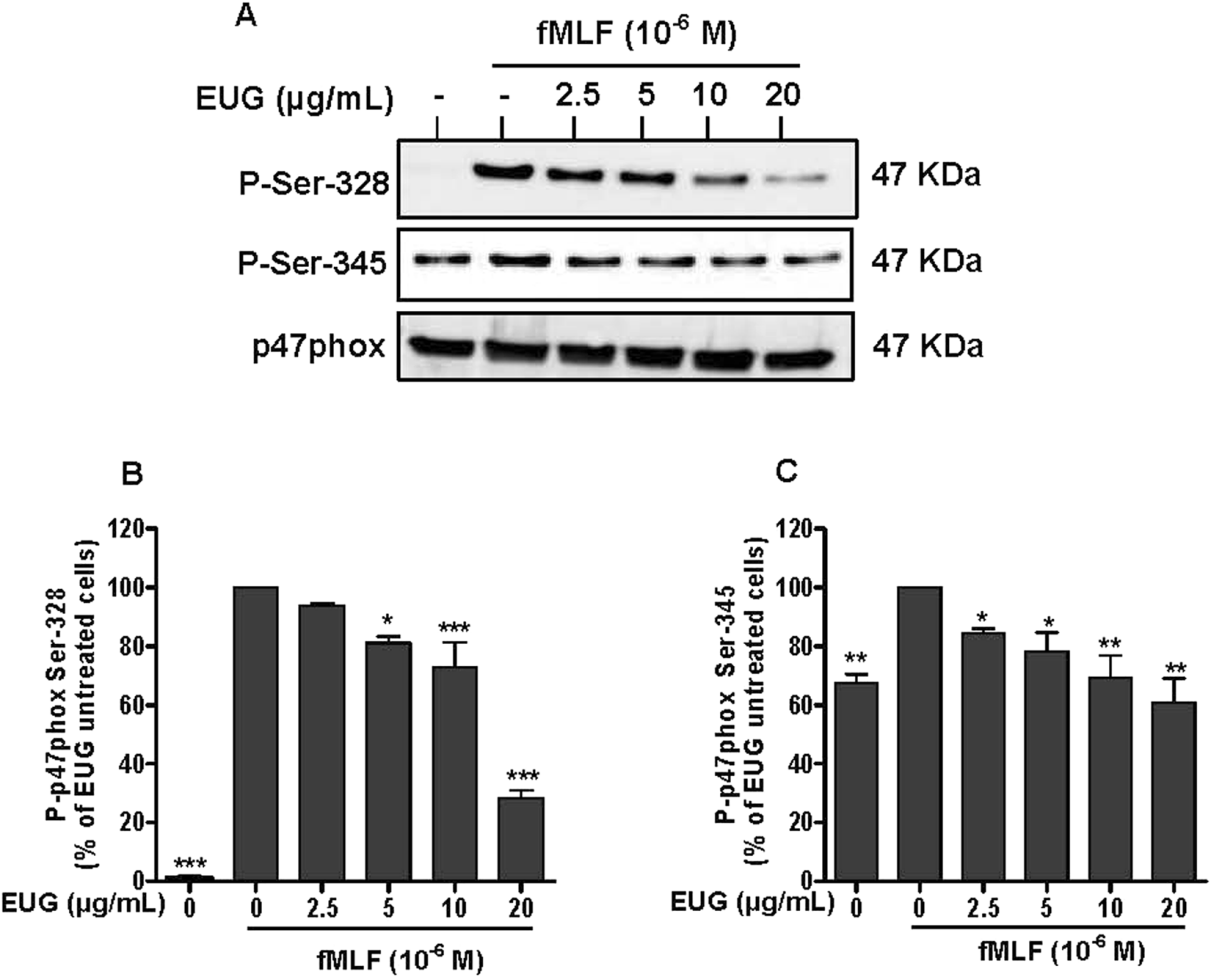
Effect of eugenol on p47phox phosphorylation in fMLF-stimulated neutrophils. Cells were incubated for 30 min at 37 °C either without or with increased concentrations of EUG (0, 2.5, 5, 10, 20 µg/mL), followed by fMLF-stimulation for 15 s. Cell lysates were analyzed by SDS-PAGE and western blot using anti-phospho-Ser-328 p47phox, anti-phospho-Ser-345 p47phox and anti-p47phox antibodies. (**A**) Western blots from different experiments were scanned and the intensity of bands was expressed relative to the total p47phoxamount. The cumulated data of phospho-Ser-328 (**B**) and phospho-Ser-345 (**C**) is shown in the histogram as percentage to control (fMLF alone 100%). Data are expressed as mean ± SEM of three or more separate experiments. p < 0.005 as compared to control (EUG untreated cells; 100%).

**Figure 4:**
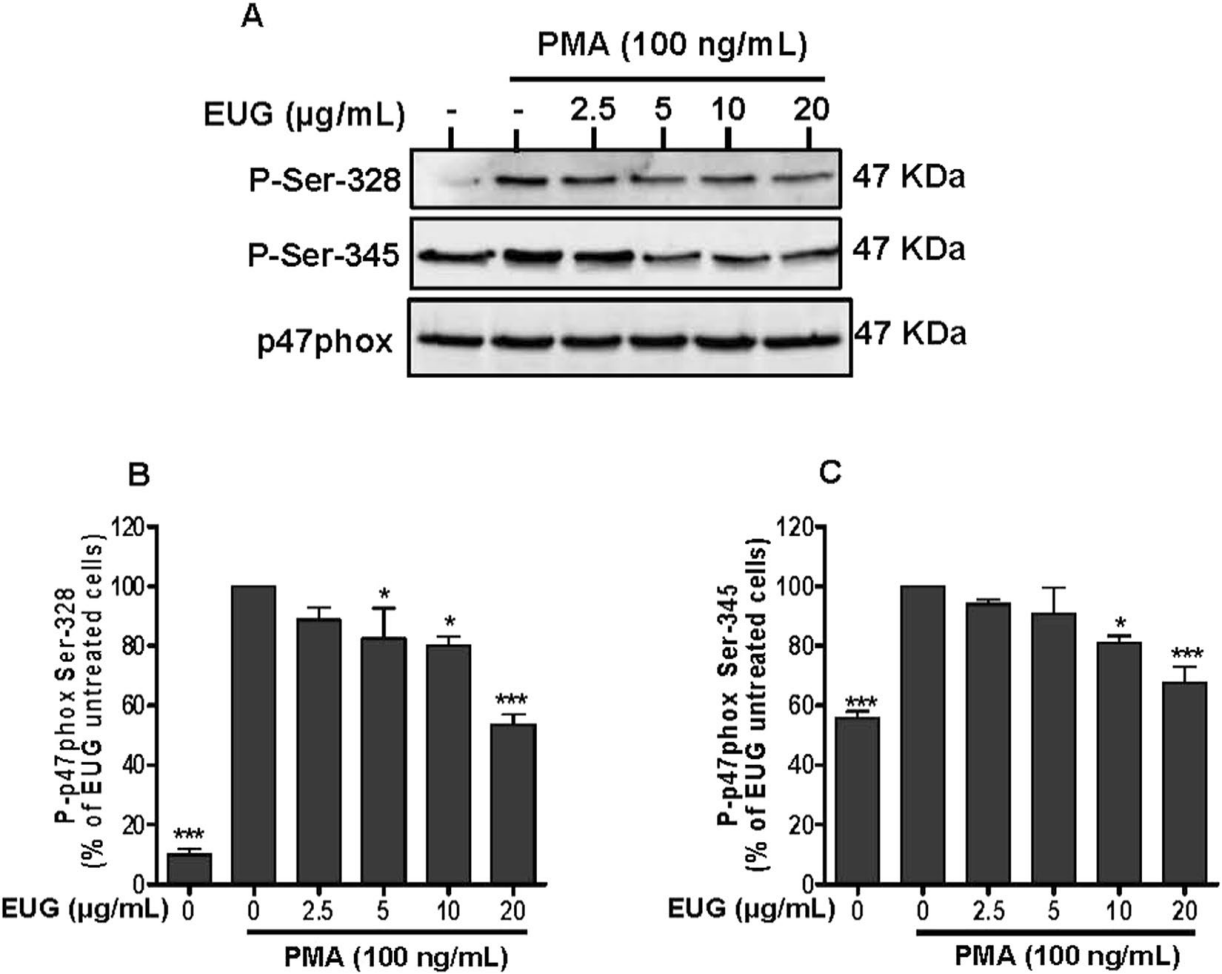
Effect of eugenol on p47phox phosphorylation in PMA-stimulated neutrophils. Cells were incubated for 30 min at 37 °C either without or with increased concentrations of EUG (0, 2.5, 5, 10, 20 µg/mL), followed by PMA-stimulation for 8 min. Cell lysates were analyzed by SDS-PAGE and western blot using anti-phospho-Ser-328p47 phox, anti-phospho-Ser-345 p47phox and anti-p47phox antibodies. **(A)** Western blots from different experiments were scanned and the intensity of bands was expressed relative to the total p47phox amount. The cumulated data of phospho-Ser-328 **(B)** and phospho-Ser-345 **(C**) is shown in the histogram as percentage to control (PMA alone 100%). Data are expressed as mean ± SEM of three or more separate experiments. p < 0.005 as compared to control (EUG untreated cells; 100%).

Superoxide anion is the precursor of other toxicreactive oxygen species (ROS) which are involved in microbial killing. ROS generation requires the activation of NADPH oxidase via phosphorylation of p47phox on various residues. Phosphorylation of serines Ser-328 and Ser-345 of p47phox subunits is critical for the activation of neutrophils NADPH oxidase and its ability to mount a potent oxidative burst in response to activating signal. For this, we examined the effect of eugenol on this process. Neutrophils were incubated with increasing concentrations of eugenol (2.5 µg/mL–20 µg/mL) and then stimulated with fMLF or PMA. The phosphorylation of p47phox residues (Ser-345, Ser-328) were detected by western blotting using specific antibodies. Results showed that eugenol decreased the phosphorylation of Ser-328 and Ser-345 in neutrophils stimulated by fMLF (Fig. 3A–C) and at lesser extent in PMA-stimulated cells (Fig. 4A–C and Supplementary file).

### Eugenol decreases translocation of p47phox from the cytosol to the membranes

**Figure 5:**
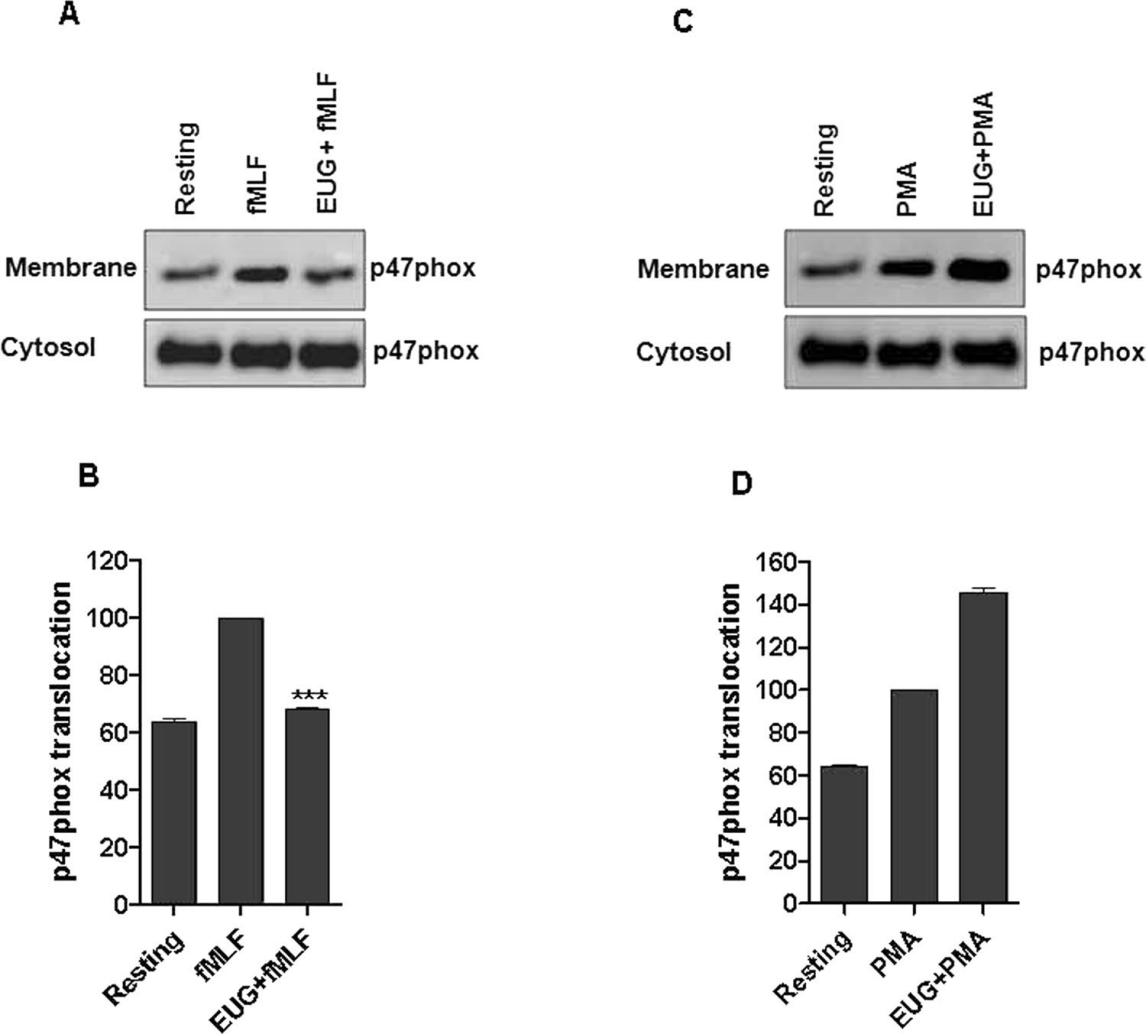
Eugenol inhibits p47phox translocation in fMLF-stimulated neutrophils. Membranes and cytosolic fractions were prepared from fMLF- and PMA-stimulated neutrophils as described in methods section. **(A**,**C)** SDS PAGE and western blotting were used to detect p47phox amounts in the sections. (**B**,**D**) Bands were quantified using Image J softwere. Values are expressed as ±SEM of three separate experiments.

The phosphorylation of p47phox on several serines induces its translocation from the cytosolic compartment to the membranes a keyprocess for NADPH oxidase activation. We thus examined the effect of eugenol on this process. Neutrophils were incubated with eugenol at concentration of 20 µg/mLand then stimulated with fMLF or PMA, neutrophils were lysed and cytosol and membranes were separated by ultracentrifugation. The translocation of p47phox was detected by western blotting using specific antibodies. Results showed that eugenol decreased significantly the translocation of p47phox subunit in neutrophils stimulated by fMLF (Fig. 5A,B) without having an effect on PMA-stimulated cells (Fig. 5C,D and Supplementary file). To the best of our knowledge this is the first report describing the effect of eugenol on p47phox translocation.

### Eugenol strongly inhibits ERK1/2 phosphorylation with less effect on p38MAPK phosphorylation

**Figure 6:**
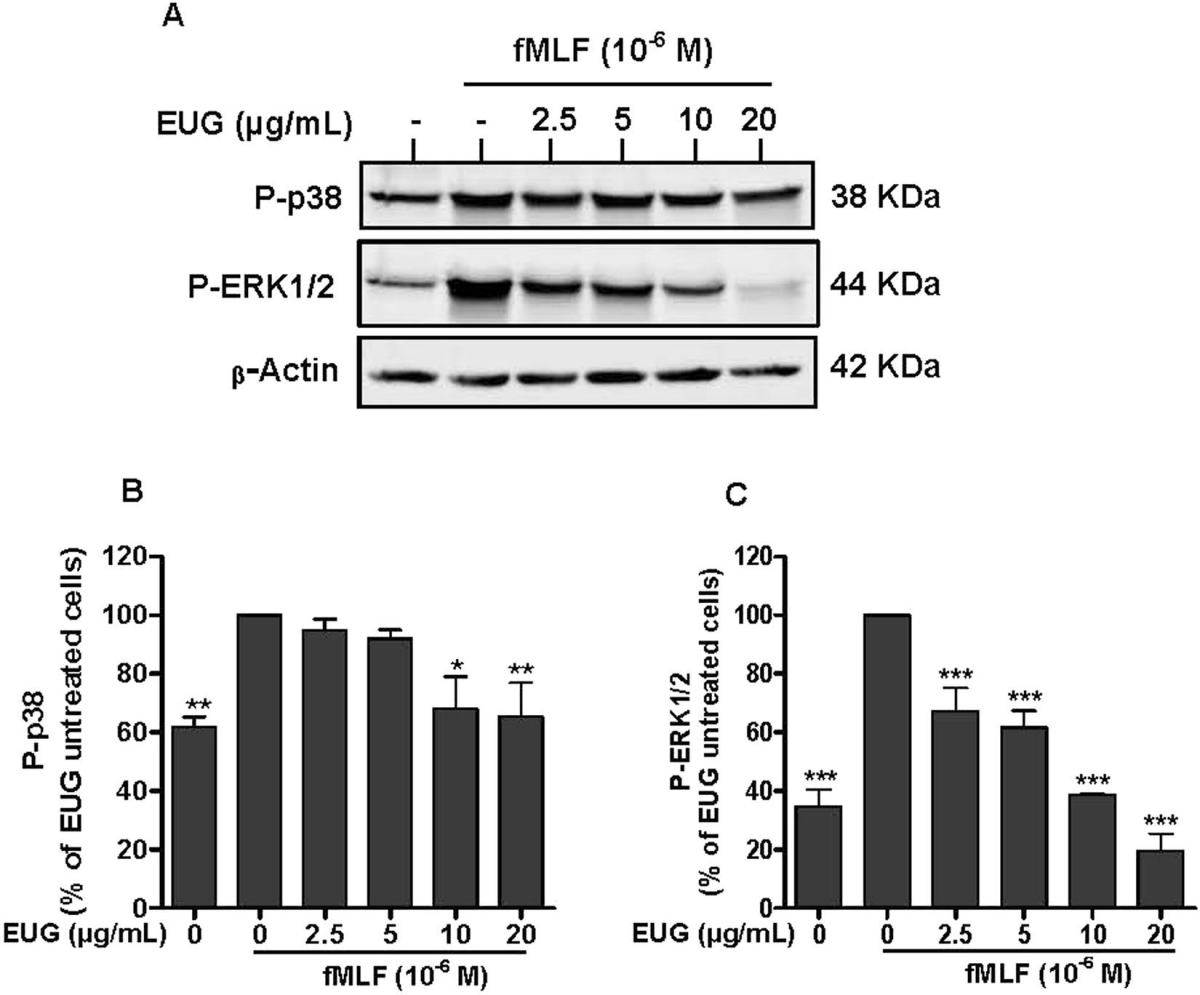
Effect of eugenol on MAP Kinases activation in fMLF-stimulated neutrophils. Cells were incubated for 30 min at 37 °C either without or with increased concentrations of EUG (0, 2.5, 5, 10, 20 µg/mL), followed by fMLF-stimulation for 15 s. Cells lysates were analyzed by SDS-PAGE and western blot using anti-phospho-ERK 1/2, anti-phospho-p38 and anti-β-actine antibodies. **(A)** Western blots from different experiments were scanned and the intensity of bands was expressed relative to β-actine amount. The cumulated data of phospho-p38 **(B)** and phospho-ERK1/2 (**C**) is shown in the histogram as percentage to control (fMLF alone 100%). Data are expressed as mean ± SEM of three or more separate experiments. p < 0.005 as compared to control (EUG untreated cells; 100%).

**Figure 7:**
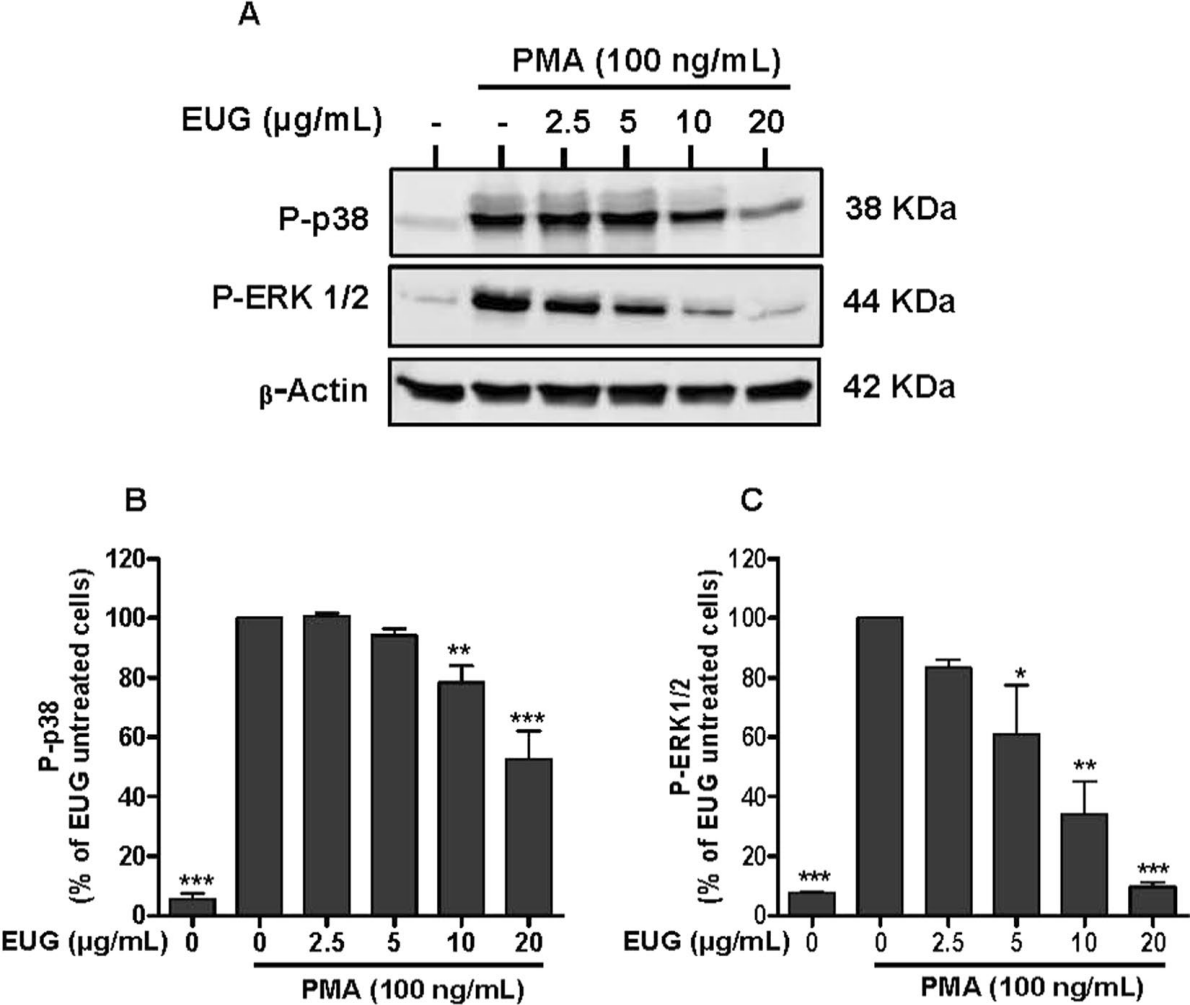
Effect of eugenol on MAP Kinases activation in PMA-stimulated neutrophils. Cells were incubated for 30 min at 37 °C either without or with increased concentrations of EUG (0, 2.5, 5, 10, 20 µg/mL), followed by PMA-stimulation for 8 min. Cell lysates were analyzed by SDS-PAGE and western blot using anti-phospho-ERK 1/2, anti-phospho-p38 and anti-β-actine antibodies. **(A)** Western blots from different experiments were scanned and the intensity of bands was expressed relative to β-actine amount. The cumulated data of phospho-p38 **(B)** and phospho-ERK1/2 **(C)** is shown in the histogram as percentage to control (PMA alone 100%). Data are expressed as mean ± SEM of three or more separate experiments. p < 0.005 as compared to control (EUG untreated cells; 100%).

To investigate whether the inhibition of superoxide anion generation by eugenol is mediated through modulation of MAPKs pathway, we further examined its effects on phosphorylation of ERK1/2 and p38MAPK. We found that fMLF-(Fig. 6A,B) and PMA-(Fig. 7A,B) induced phosphorylation of ERK in stimulated neutrophils was decreased by pretreatment with eugenol at different concentrations. The inhibitory effect is dose dependent and significant at low dose of 2.5 µg/mL. The effect of eugenolon p38 MAPK (Figs. 6C and 7C) was significantonlyat high concentrations (10 µg/mL and 20 µg/mL).

### Eugenol inhibits MEK1/2 and Raf phosphorylation

**Figure 8:**
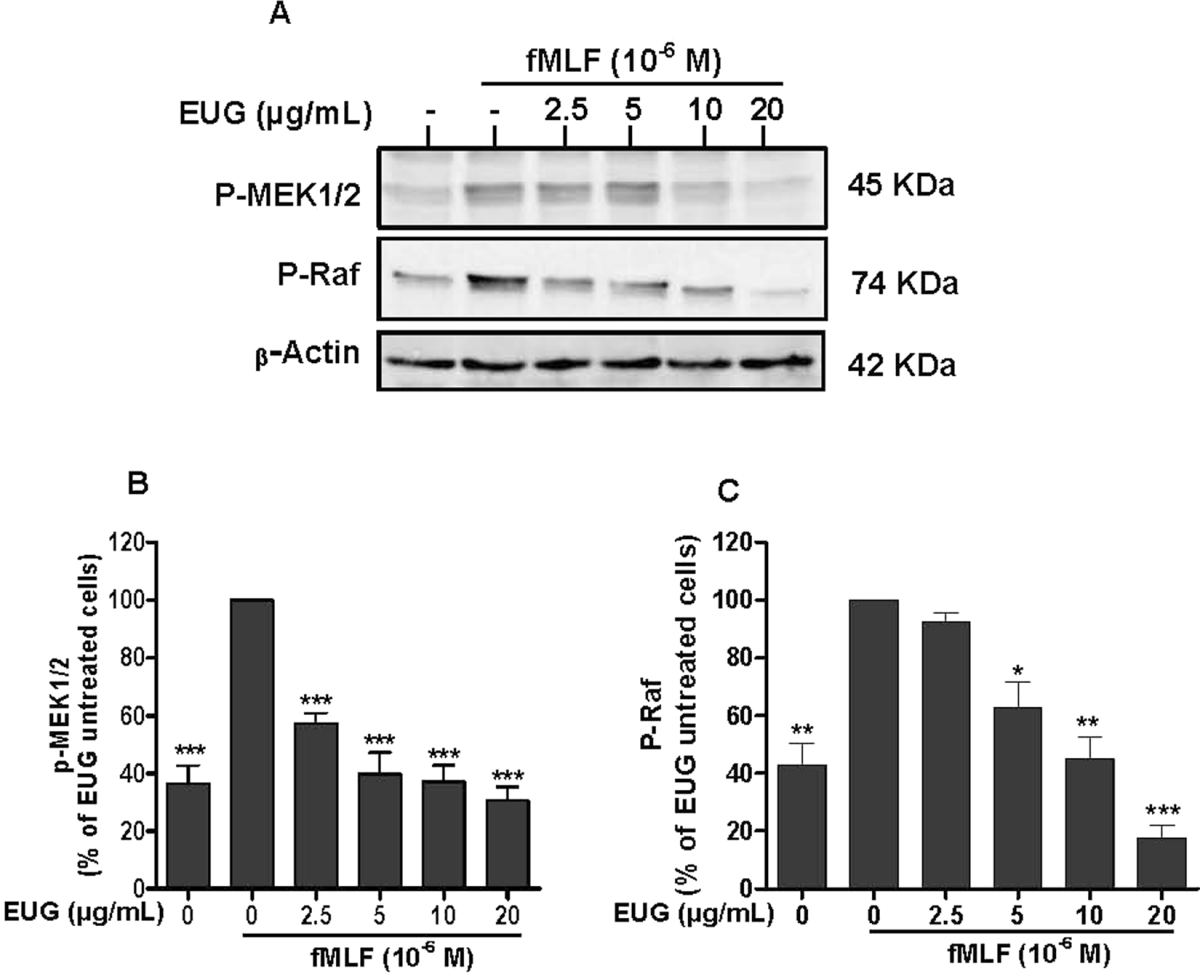
Effect of eugenol on MEK1/2 and Raf phosphorylation in fMLF-stimulated neutrophils. Cells were incubated for 30 min at 37 °C either without or with increased concentrations of EUG (0, 2.5, 5, 10, 20 µg/mL), followed by fMLF-stimulation for 15 s. Cell lysates were analyzed by SDS-PAGE and western blot using anti-phospho-MEK 1/2, anti-phospho-Raf and anti-β-actine antibodies. (**A**) Western blots from different experiments were scanned and the intensity of bands was expressed relative to β-actine amount. The cumulated data of phospho-MEK 1/2 **(B)** and phospho-Raf (**C**) is shown in the histogram as percentage to control (FMLF alone 100%). Data are expressed as mean ± SEM of three or more separate experiments. p < 0.005 as compared to control (EUG untreated cells; 100%).

**Figure 9:**
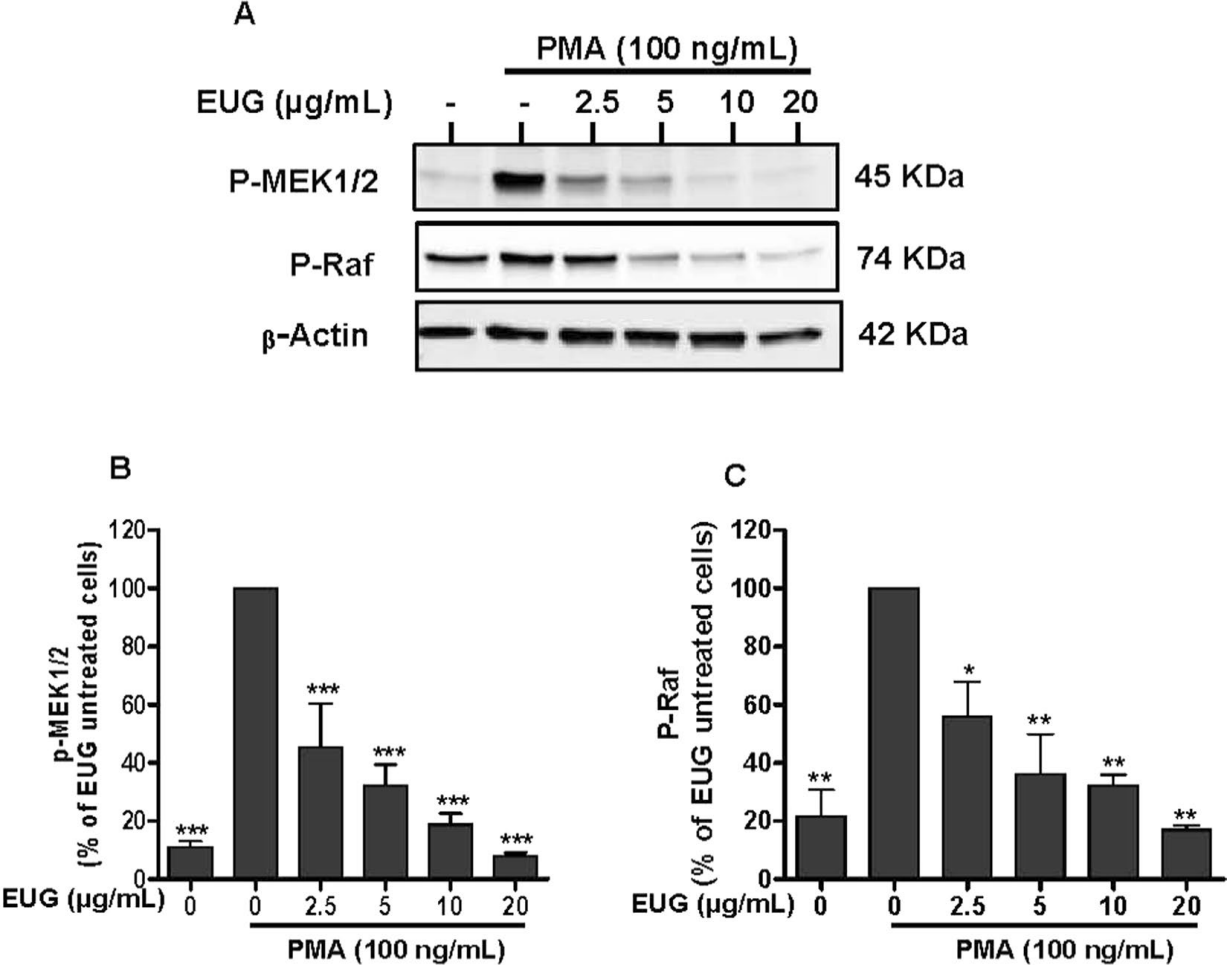
Effect of eugenol on MEK1/2 and Raf phosphorylation in PMA-stimulated neutrophils. Cells were incubated for 30 min at 37 °C either without or with increased concentrations of EUG (0, 2.5, 5, 10, 20 µg/mL), followed by PMA-stimulation for 8 min. Cell lysates were analyzed by SDS-PAGE and western blot using anti-phospho-MEK 1/2, anti-phospho-Raf and anti-β-actine antibodies. (**A**) Western blots from different experiments were scanned and the intensity of bands was expressed relative to β-actine amount. The cumulated data of phospho-MEK 1/2 **(B)** and phospho-Raf **(C)** is shown in the histogram as percentage to control (PMA alone 100%). Data are expressed as mean ± SEM of three or more separate experiments. p < 0.005 as compared to control (EUG untreated cells; 100%).

As eugenol inhibited ERK1/2 phosphorylation, we investigated the effect on the phosphorylation of the upstream kinases MEK1/2 and Raf in human neutrophils using SDS-PAGE and Western Blot. Results show that compared with the control, the phosphorylation levels of MEK1/2 and Raf were dramatically decreased in Eugenol-treated neutrophils. The inhibition was significantly dose dependent from a concentration of 2.5 µg/mL in fMLF- (Fig. 8A–C) and PMA- (Fig. 9A–C) stimulated neutrophils.

## Discussion

Neutrophils are the most abundant white cells in humans. They are the first line of host defense in organisms. During infection, they play a key role in destroying infectious agent by oxidative and non oxidative mechanisms^19^. Excessive ROS production and accumulation seem to play a pivotal role in tissue injury and development of inflammatory diseases^20^. Thus, the inhibition of ROS over production and neutrophils hyperactivation represent a major research interest. Phytochemicals such as flavonoids, present in several plants, have been reported to have antioxidant and immunomodulatory propreties^21^. Clove (*Syzygiumaromaticum*) a well known spice, is characterized by several biological activities especially antioxidant and anti-inflammatory effects. Its biological activities have been attributed to eugenol (EUG), the most abundant constituent of essential oil (45–90%) of *Syzygium aromaticum*^22^. Several studies have recently demonstratedthe importance of eugenol as abioactive molecule with various pharmacological properties. In this work, we showed that eugenol inhibits significantly and dose dependently the strong fMLF-induced superoxide anion production by human neutrophils with IC50 of 5 µg/mL. The inhibitory effect of eugenol on superoxide anion production by PMA-stimulated neutrophils, was observed with 20 µg/mL (p < 0.05), the highest concentration used in this study. The difference in the inhibitory effect of eugenol on fMLF- and PMA-stimulated superoxide anion production could be explained by the different mechanisms of neutrophil activation. Thus, we can suggest that low concentrations of eugenol had no effect on PKC activity. Our findings are in agreement withKim *et al*., whostudied the antioxidant activity of eugenolusing ABTS and DPPH free radicals. At concentration of 20 µg/mL, eugenol performed the elimination of 76.9% and 90.8% of ABTS and DPPH free radicals, respectively^23^. Superoxide anion, the precursor of other reactive oxygen species, is frequently produced byactivated neutrophils through the NADPH oxidase complex^24^. The activation of this enzyme requires the phosphorylation of its cytosolic component p47phox on serines located between Ser-303 and Ser-379^25^. It is known that stimulation of neutrophils by high concentrations of the chemotactic peptide fMLF or by the PKC agonist PMA induces complete phosphorylation of p47phox^14^. In the current study, we haveshown that eugenol, at concentration of 5; 10 and 20 µg/mL, reduceddose dependentlythe phosphorylation of both Ser-328 and Ser-345 residues in neutrophils stimulated by fMLF and PMA. A pronounced inhibition was observed withSer-345 for both fMLF and PMA studies. Since, the same amount of p47phox was loaded into each well, these results suggest that the inhibition of superoxide anion production by stimulated neutrophils is due in part to reduced phosphorylation of p47phox. Moreover, it is known that the NADPH oxidase is tightly regulated by the translocation of p47phox from the cytosol to the membranes, we demonstrated that eugenol at 20 µg/mL reduces the translocation of p47phox to neutrophils membranes and prevented the assembly of a functional NADPH oxidase enzyme. To the best of our knowledge this is the first report describing the effect of eugenol on p47phox translocationThese results all together suggest that eugenol reduce the phosphorylation of p47phox on specific serines, and consequently reduce its translocation to the membrane and the assembly of active NADPH oxidase

The activation of NADPH oxidase via the phosphorylation of p47phox subunits is performed by kinases such as protein kinases C (PKC) andMitogen-activated protein kinases (MAPK)^26^. MAPKinaseis a family of Mitogen-activated protein kinases implicated in many biological processes. Extracellular signal-regulated kinase (ERK) 1/2, c-Jun N-terminal kinase (JNK) and mitogen-activated protein kinases p38 are the most studied families of MAPKs^27^. ERK1/2 and p38 MAPK are activated in neutrophils in response to a wide variety of pro-inflammatory stimuli including growth factors, phorbol esters, fMLF and cytokines. They are implicated in the respiratory burst, adherence, exocytosis and priming^28^. Ourresult showed that eugenol inhibits phosphorylation of ERK1/2 dose dependently as low as 2.5 µg/mL. However, the phosphorylation of p38 MAPK was decreased significantly at the two highest tested concentrations, 10 µg/mL and 20 µg/mL. p38 MAPK and ERK1/2 are required for the phosphorylation of p47phox on Ser-345. This phosphorylation is critical for NADPH oxidase hyperactivation and priming^29^. On the basis of our observations, we can suggest that the inhibition of phosphorylation of Ser-345 and Ser-328 is due to the decrease of MAPKs phosphorylation and activities. Similar data were reported by Deepak *et al*., in a model of osteoclastogenesis where by a pretreatment of RAW264.7 macrophages with 200 µM of eugenol, for 4 hours, inhibited the phosphorylation of ERK, JNK and p38^30^. Another *in vitro* study showed that two synthetic derivates of eugenol, glyceryl-isoeugenol and eugenolol, attenuated LPS-induced phosphorylation of ERK, p38 MAPK and JNK in macrophages^31^. Phosphorylation and activation of ERKs is accomplished by MEK1 and MEK2, two relevant specific MAPKs, which are, to date, the only validated physiologically substrates of Raf serine/threonine kinases^32^. Several studies reported the implication of Raf and MEK in many diseases essentially in cancer development^33^. For this, Target-based therapies are considered as advanced approaches of cancer treatment and there isstrong interest in developing pharmacological inhibitors of Raf and MEK as a means to block ERK signaling^34^. Our results show clearly that eugenol inhibits the phosphorylation of Raf and MEK in fMLF and PMA stimulated neutrophils. These results suggest that eugenol could be a promising inhibitor of Raf/MEK/ERK cascade.

Taken together, this study demonstrates that eugenol may suppress fMLF-mediated production of superoxide anion by down regulating the activity of NADPH oxidase via the inhibition of Raf/MEK/ERK cascade and the phosphorylation of p47phox on activators residues (Ser-345 and Ser-328). Our findings demonstrate the molecular basis for the antioxidant proprieties of eugenol and show its potential therapeutic role in the treatment of oxidative stress-related diseases.

## Methods

### Reagents

Eugenol, Hank’s Balanced Salt Solution (HBSS), Phosphate Buffered Saline (PBS), Phorbolmyrisate acetate (PMA), formyl-methionyl-leucyl-phenylalanine (fMLF), mouse monoclonal anti-β-actin antibody, phosphatase and proteases inhibitors were from Sigma Aldrich (Saint Quentin Falavier, France). Dextran T500 and Ficoll were purchased from GE healthcare (Orsay, France). SDS-PAGE and western blot reagents were from Bio-Rad Laboratories (Hercules, CA, USA). Anti-phospho-Raf, Anti-phospho-MEK1/2, Anti-phospho-p38 and Anti-phospho-ERK 1/2 antibodies were from cell signaling Technology (Boston, MA, USA). The anti-phospho-p47phox antibodies (Ser-328 and Ser-345) were generated by our lab as previously described^35^. HRP-conjugated goat anti-rabbit, HRP-conjugated goat anti-mouse, AP-conjugated goat anti-rabbit antibodies and ECL (enhanced chemiluminescence) reagent were from Santa Cruz Biotechnology Inc (Heidelberg, Germany).

### Ethics statement and neutrophils isolation from human blood

The clinical Research Committee at the Xavier Bichat hospital (Paris, France) approved all methods, performed in accordance with the internationals guidelines and regulations. Venous blood was collected from healthy volunteersafter obtaining written informed consent. The density gradient separation method was used to isolate human neutrophils from whole blood using 2% Dextran (T500) and ficoll centrifugation^36^. Red blood cells were removed by hypotonic lysis. To prevent activation, isolated neutrophils were resuspended in HBSS without Ca^2+^/Mg^2+^. A cell count was performed and cell viability was determined using the trypan blue exclusion method.

### Cytotoxicity of eugenol

Isolated neutrophils (1 × 10^6^ cells) were incubated in absence or presence of increasing concentrations of eugenol (0–100 µg/mL) for 30 min at 37 °C. The trypan blue dye test is used to determine the number of viable cells. Results are presented as the percentage of viable cells compared to untreated cells.

### Measurement of superoxide anion production

Neutrophils (1 × 10^6^ cells) were suspended in 1 mL of HBSS containing 1 mg/mL cytochrome c in the presence or absence of increasing concentration of eugenol (2.5 µg/mL–20 µg/mL). After incubation in the thermostated chamber of a spectrophotometer (Uvikon, Villebon, France) for 30 min at 37 °C, cells were stimulated with fMLF (10^−6^ M) or PMA (100 ng/mL). Changes in absorbance were measured at 550 nm for 10 min.

### p47phox translocation to plasma membranes

The experiment was performed as previously described^37^. Isolated neutrophils (50 × 10^6^ per mL) were incubated for 30 min at 37 °C in the absence or presence ofeugenol at 20 µg/mL. Cells were then stimulated by fMLF (10^−6^ M) or PMA (100 ng/mL). The stimulation was stopped by addition of ice cold PBS, followed by centrifugation at 400 × g for 10 min. The cell pellet was resuspended and sonicated on icein the described relaxation buffer^37^, then centrifuged at 400 × g for 10 min. The lysates were then ultracentrifuged at 150 000 × g for 45 min at 4° on a sucrose gradient. The cytosolic and membrane fractions were collected and washed in the same lysis buffer, denaturated in Laemmli sample buffer and storedat −80 °C until use.

### SDS-PAGE and western blotting analysis

Human neutrophils were treated or not with increasing concentrations of eugenol (2.5 µg/mL–20 µg/mL) for 30 min at 37 °C and subsequently stimulated with fMLF (10^−6^ M for 15 seconds) or PMA (100 ng/mL for 8 min). The reaction was stopped by adding 5× concentrating Laemmli sample buffer supplemented by 12.5 mM Na_3_VO_4_, 25 mMNaF, 6.25 mM p-NPP, 12.5 mM EDTA, 12.5 mM EGTA, 50 µg/mL pepstatin and 50 µg/mL aprotinin. Samples were vortexed immediately and denaturated for 3 min at 100 °C. Following sonication, the lysates were subjected to sodium dodecyl-sulfate polyacrylamide (SDS-PAGE) (10%) to separate the proteins. The proteins separated through electrophoresis were transferred onto nitrocellulose membranes followed by blocking the membranes with 5% non fat dry milk in a mixture of tris-bufferd saline and tween-20. The membranes were incubated overnight at 4 °C in solution containing specific relevant primary antibodies; anti-phospho-S328-p47phox (1:2500), anti-phospho-S345-p47phox (1:10000), anti-phospho-Raf (1:1000), anti-phospho-MEK1/2 (1:1000), anti-phospho-ERK1/2 (1/2000), anti-phospho-p38 (1:2000) and p47phox (1:5000), following by incubation in horseradish peroxidase conjugated secondary antibodies (Santa Cruz, Heidelberg, Germany). An enhanced chemilumiscence solution ECL (Santa Cruz Biotechnology Inc. Heidelberg, Germany) was added to the membranes and proteins were detected and quantified using the biospectrum imaging system (Amersham Imager 600).

### Statistical analysis

All experiments were performed in at least triplicates per group and data are reported as mean ± SEM. One way analysis of variance followed by Newman-Keuls multiple comparisons test was used for statistical analysis. p values less than 0.05 were considered as statistically significant.

## Supplementary information

Related Manuscript File

## Data Availability

All relevant data are within the paper.
