# Supplementary material for: Eugenol prevents fMLF-induced superoxide anion production in human neutrophils by inhibiting ERK1/2 signaling pathway and p47phox phosphorylation
Source: Sci Rep. 2019 Dec 6;9:18540. doi: 10.1038/s41598-019-55043-8 (PMC6898361; doi:10.1038/s41598-019-55043-8)

**Supplementery information**

**Eugenol prevents fMLF-induced superoxide anion production in human neutrophils by inhibiting ERK1/2 signaling pathway and p47phox phosphorylation**

Amina Chniguir^a,b^, Coralie Pintard^c,d^, Dan Liu^c,d^, Pham My-Chan Dang^c,d^, Jamel El-Benna^c,d^ & Rafik Bachoual* ^a,b^

^a^Faculty of Sciences of Gabes; University of Gabes, Tunisia

^b^Laboratory of Plant Improvement and Valorization of Agroresources, National School of Engineering of Sfax, Sfax, Tunisia.

^c^INSERM U1149, CNRS ERL8252 Inflammation Research Center, Paris, France.

^d^University of Paris Diderot, Sorbonne Paris City, Inflamex Laboratories, Faculty of Medicine, Xavier Bichat, Paris, France.

**Raw Data (Fig. 3)**


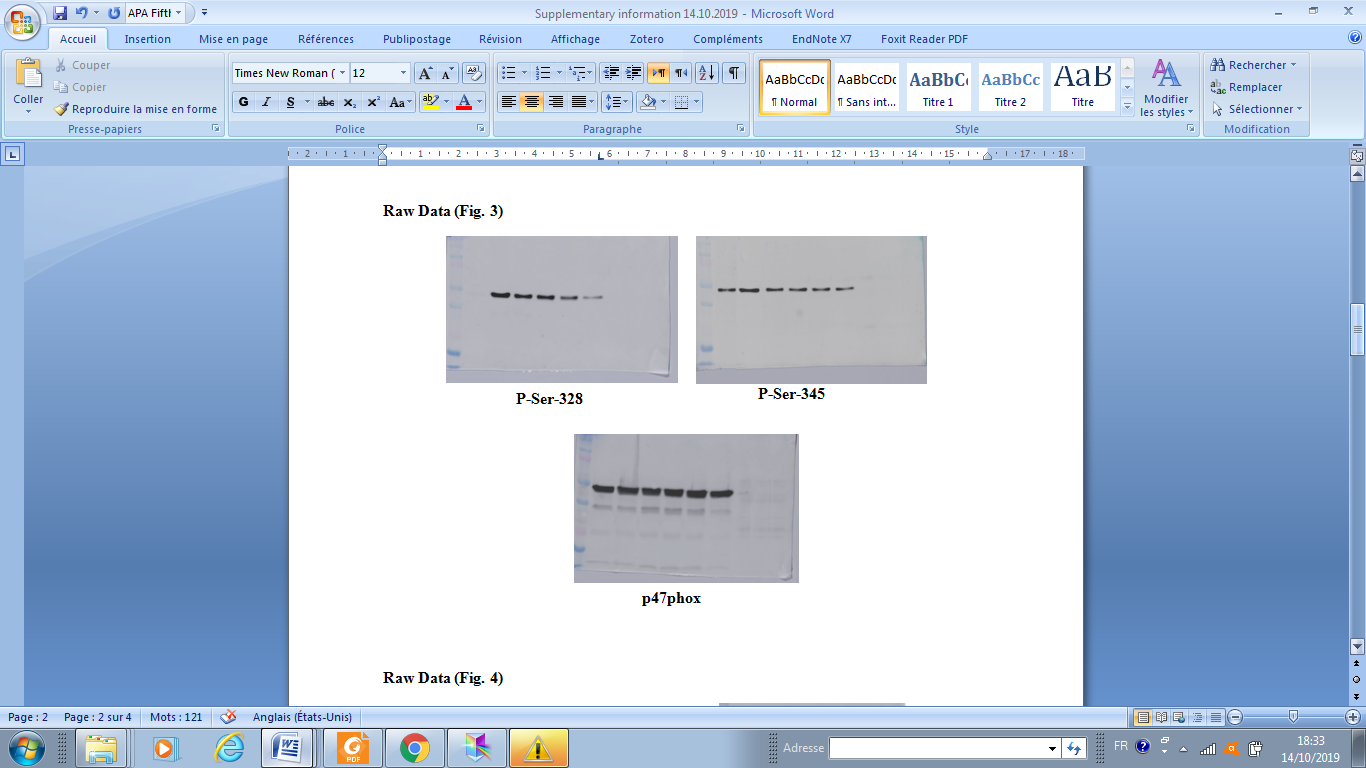


**Raw Data (Fig. 4)**


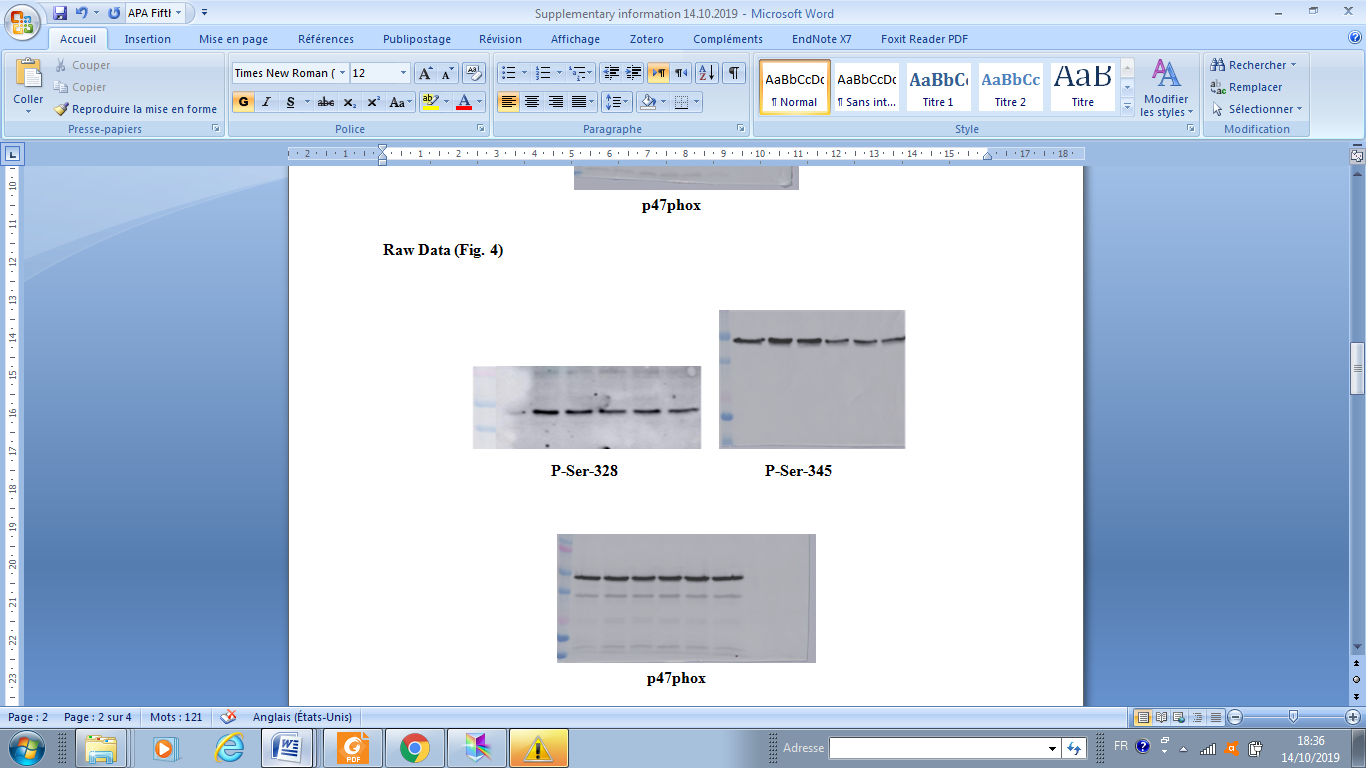


**Raw Data (Fig. 5)**


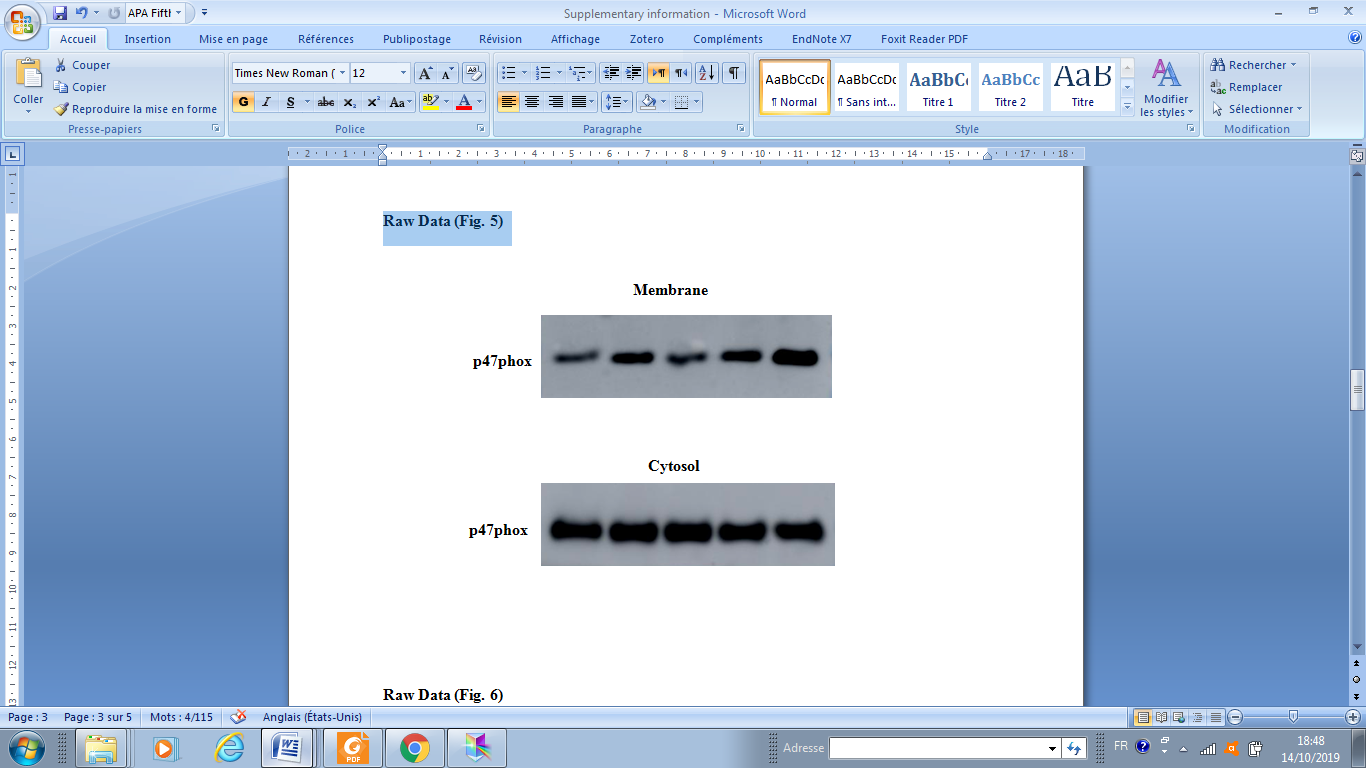


**Raw Data (Fig. 6)**


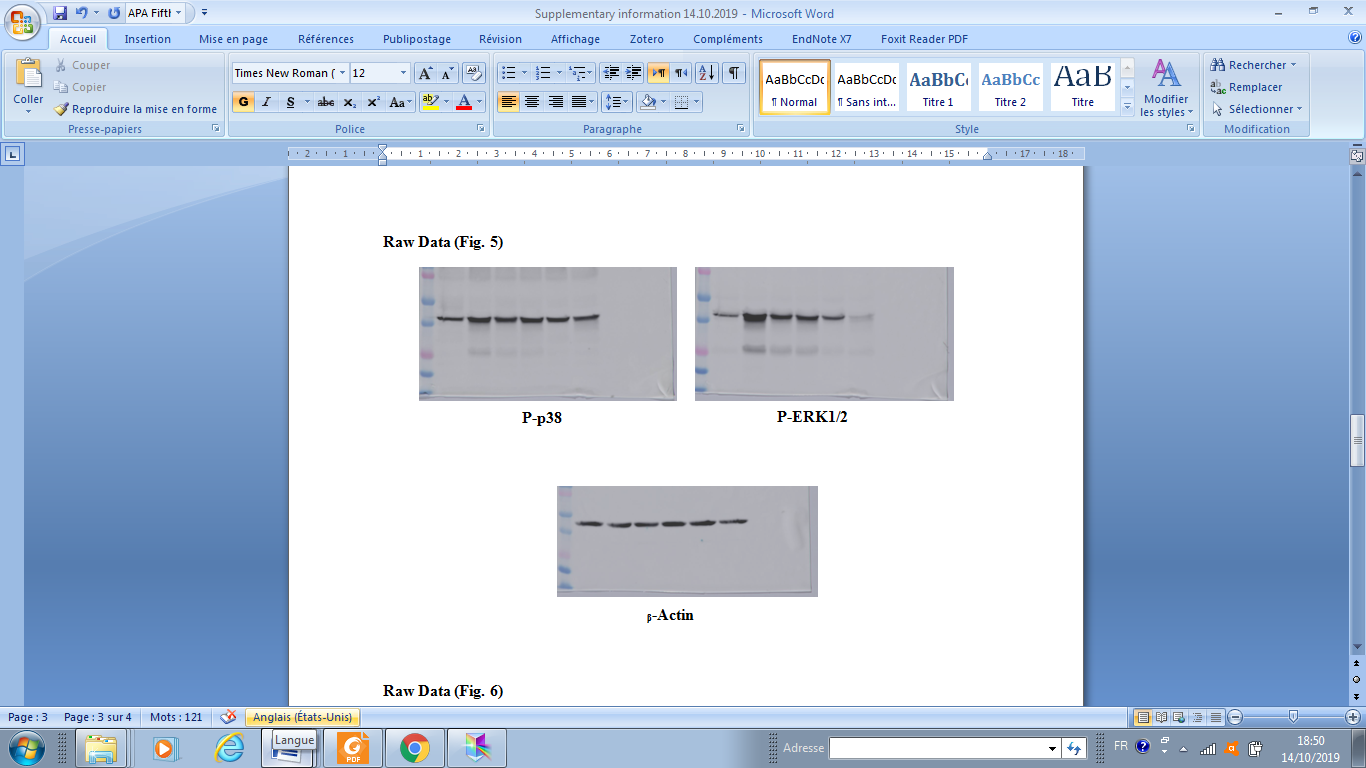


**Raw Data (Fig. 7)**


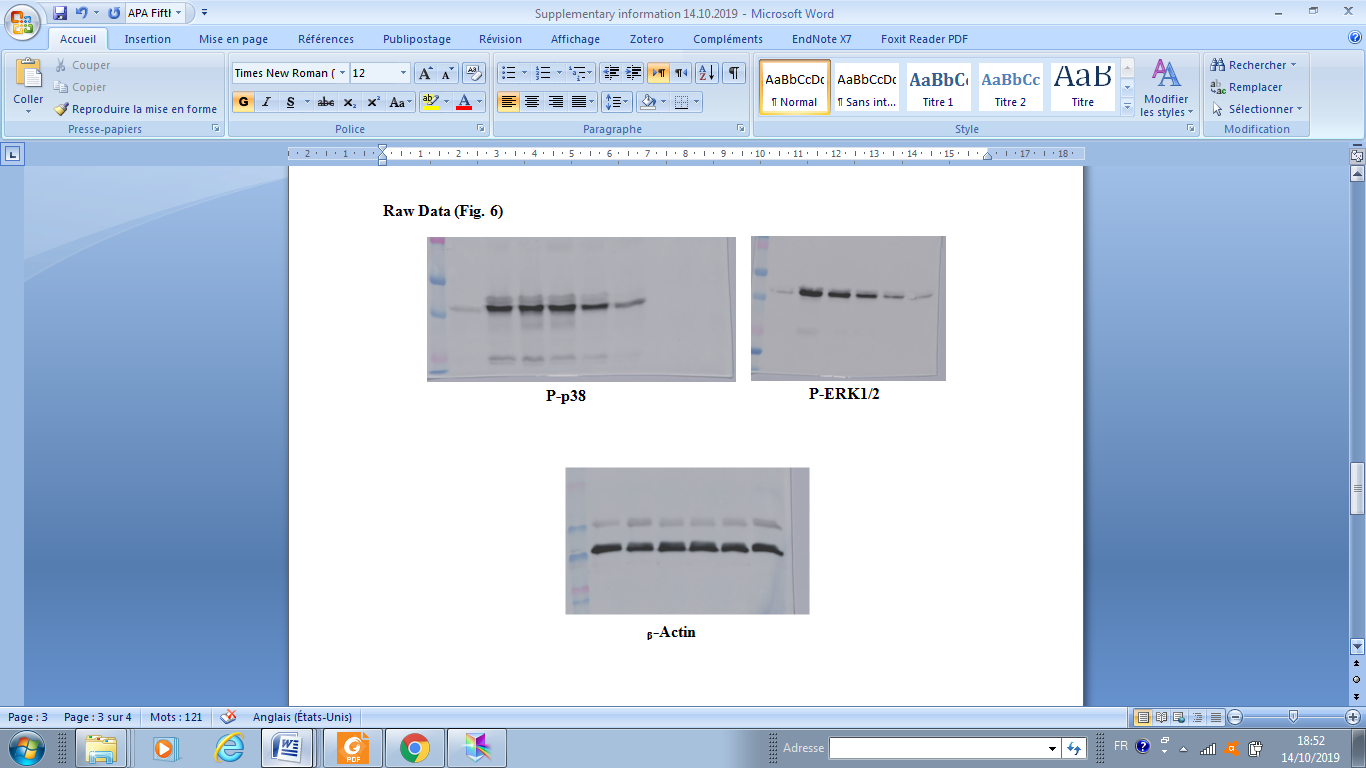


**Raw Data (Fig. 8)**


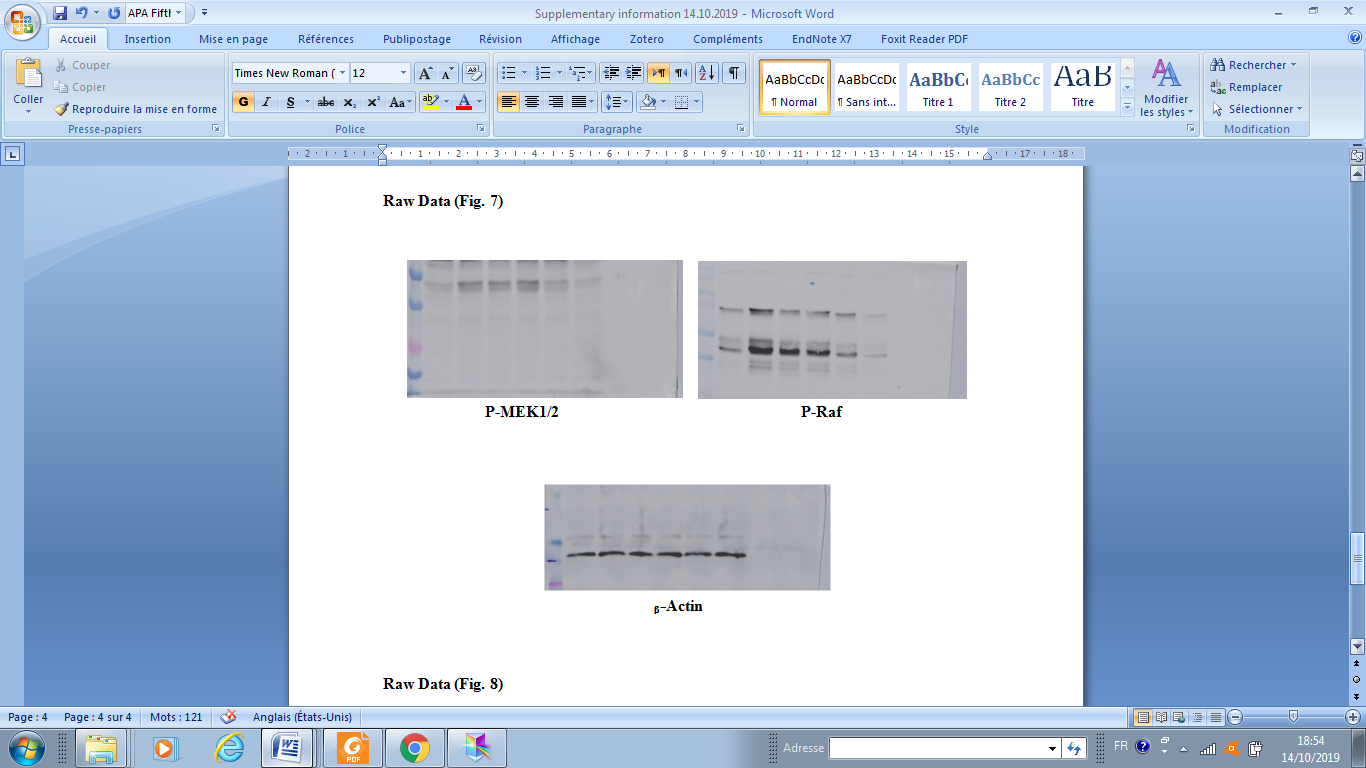


**Raw Data (Fig. 9)**


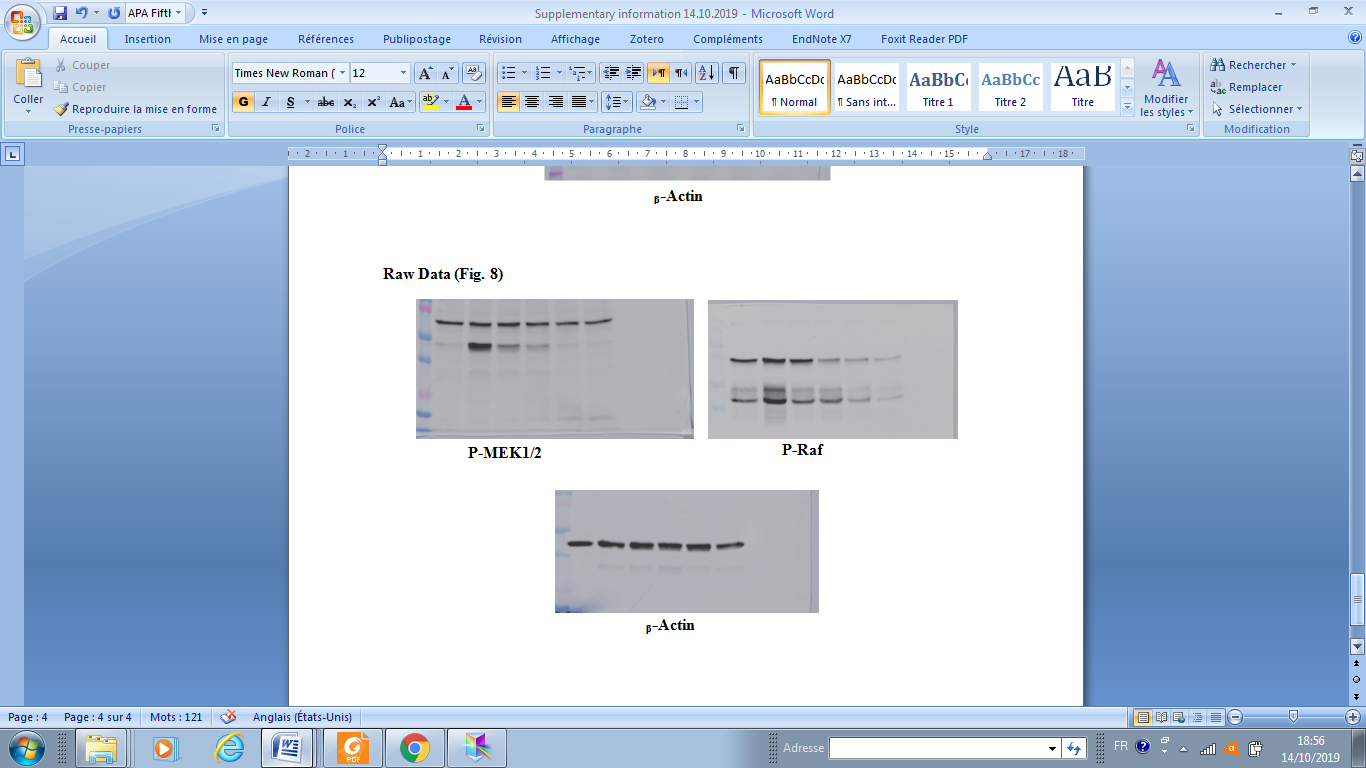

Supplement: Supplementary file 1 — Related Manuscript File [file 41598_2019_55043_MOESM1_ESM.docx]
